# Detection of intracellular IgD using flow cytometry could be a novel and supplementary method to diagnose IgD multiple myeloma

**DOI:** 10.1186/s12885-018-4562-8

**Published:** 2018-06-11

**Authors:** Wei Wang, Chun-Xia Zhang, Zhen-Ling Li, Ming Gong, Yi-Gai Ma

**Affiliations:** Hematology Department, China-Japan Friendship Hospital, Ying-Hua-Yuan East Street, No. 2, Beijing, 100029 China

**Keywords:** Multiple myeloma, IgD, Flow cytometry, Cytoplasmic IgD, Diagnosis

## Abstract

**Background:**

We examined whether detecting the heavy chain of cytoplasmic immunoglobulin D (IgD) by flow cytometry could be used as a supplemental method to diagnose IgD multiple myeloma (MM).

**Methods:**

Bone marrow (BM) samples of thirty-five patients with MM were collected. Five of them were IgD MM, the rest of thirty were other subtypes of MM. Antibodies to four types of heavy chains of immunoglobulin (e.g., IgA, IgG, IgM, and IgD) were analyzed by flow cytometry in each patient’s BM sample.

**Results:**

The five IgD MM patients were all positive for cytoplasmic IgD. The percentage of IgD positive MM cells among nucleated cells varied from 0.4 to 12.9%. Cytoplasmic IgG was positive in eight patients with IgG MM (*n* = 9); cytoplasmic IgA was positive in all patients with IgA MM (*n* = 10); cytoplasmic IgM was positive in one patient with IgM MM (n = 1). No heavy chain was detected in light chain MM (n = 9) and non-secretory subtype (*n* = 1).

**Conclusions:**

Detection of cytoplasmic IgD by flow cytometry is a convenient, sensitive and supplemental method to diagnose IgD MM.

## Background

Immunoglobulin D (IgD) multiple myeloma (MM) comprises 1 to 2% of all MM cases [1, 2]. It is characterized by occurrence in relatively young patients, osteolytic lesions, extramedullary involvement, amyloidosis, renal failure, and a poor prognosis [2–6]. Diagnosis of IgD MM is difficult because IgD can present minimal or even undetectable M-protein spikes via serum protein electrophoresis (SPEP) [2, 7]. Thus, many cases present as hypogammaglobulinemia or have normal SPEP results. This could lead to misdiagnoses of patients in this subgroup [8, 9].

In this research, we describe a new method to detect cytoplasmic IgD in malignant plasma cells using multiple parameter flow cytometry as a supplementary method to diagnose IgD MM.

## Methods

### Patients

The study was approved by the Ethical Committee of the China-Japan Friendship Hospital (Reference Number: 81300450). It was conducted in accordance with the ethical standards described in the 1964 Declaration of Helsinki and its later amendments or comparable ethical standards. Written informed consents for participation in the study were obtained from participants. Bone marrow (BM) samples from ten patients with newly diagnosed MM and twenty-five MM patients after chemotherapy were collected from February 2016 to November 2017. The median age was 66 years (range, 45–81 years). All patients were diagnosed based on the International Myeloma Working Group (IMWG) diagnostic criteria [10].

### Monoclonal immunoglobulin detection

Monoclonal immunoglobulins were detected by SPEP, urine protein electrophoresis (UPEP), serum and urine immunofixation electrophoresis (IFE), and serum and urine-free light-chain (FLC) assays [11, 12]. In this study, we refer to these methods as traditional methods, and use them as the gold standard criteria to evaluate the diagnostic efficiency of flow cytometry as for detecting the expression of cytoplasmic immunoglobulin heavy chain.

### Flow cytometry analysis

For flow cytometry analysis, BM samples from ten patients before chemotherapy and samples from twenty-five patients after chemotherapy were evaluated.

The following monoclonal antibodies (mAbs) were purchased from DAKO: anti-IgA FITC (F0188), anti-IgG FITC (F0185), anti-IgM PE (R5111), anti-Kappa PE (R0436), and anti-Lambda FITC (F0435). Anti-IgD PE (348204) was purchased from Biolegend. The following mAbs were purchased from Becton Dickinson (BD): anti-CD19 V450 (560353), anti-CD20 APC-H7 (560734), anti-CD38 PC7 (560677), anti-CD45 V500 (560777), and anti-CD138 APC (566050). The following mAb panels were used in the study: CD19 V450/CD45 V500/ cytoplasm IgG FITC/ cytoplasm IgM PE/CD38 PC7/CD138 APC/CD20 APC-H7; CD19 V450/CD45 V500/ cytoplasm IgA FITC/cytoplasm IgD PE/CD38 PC7/CD138 APC/CD20 APC-H7; CD19 V450/CD45 V500/cytoplasm Lambda FITC/cytoplasm Kappa PE/CD38 PC7/CD138 APC/CD20 APC-H7. An IntraStain kit (K2311, DAKO) was used for intracellular staining. BD FACS Lysing Solution (349,202, BD) was used to lyse red blood cells.

BM samples were collected in heparin anticoagulant tubes; then, 5 × 10^5^ white blood cells per tube were stained and analyzed within 6 h after procurement. All samples were analyzed using FACS CantoII and Davi software (both from BD).

The gate strategy was as follows: first, we used the FCS/SSC to remove dead cells; second, we used FSC-A/FSC-H to remove cells that were clumped together; third, we delineated plasma cells on the CD38/CD138 dot plot; fourth, we used CD45 and CD19 to identify malignant plasma cells in each tube; and finally, the intracellular expression of light chains or heavy chains of immunoglobulin was detected in the malignant plasma cellular population. The phenotypes of malignant plasma cells include CD38 ^positive/dim/negative^, CD138 ^positive^, CD45 ^dim/negative^, CD19 ^negative^ [13].

### Statistical analysis

A Chi-square test was used to calculate the diagnostic coherence between flow cytometry and traditional methods (e.g., SPEP, UPEP, serum and urine IFE, serum and urine FLC assay). The statistical test was two-sided, with significance defined as *p* < 0.05. Analyses were calculated using SPSS version 17.0 (SPSS Inc., Chicago).

## Results

**Fig. 1:**
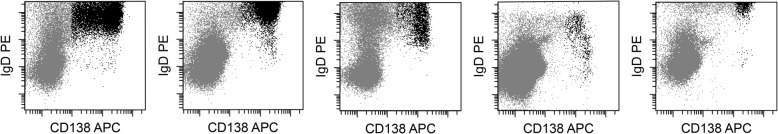
Flow cytometry results for five patients with IgD MM. Bone marrow samples from five patients with IgD MM were analyzed by flow cytometry. Expression of cytoplasmic IgD in MM cells was positive in each patient

First, we chose four patients with IgD MM after receiving chemotherapy and then detected the expression of four types of cytoplasmic immunoglobulin heavy chains in residual MM cells from their BM samples. Then, we used the same method to analyze ten BM samples from newly diagnosed MM patients and twenty-one samples from MM patients after chemotherapy. Five IgD MM patients diagnosed by traditional methods were all positive for cytoplasmic IgD. The clinical information of the five patients is listed in Table 1. The percentage of MM cells among nucleated cells varied from 0.4 to 12.9%. The flow cytometry results of the five patients are shown in Fig. 1.

**Table 1:** Clinical information of patients with IgD MM before chemotherapy

Patient No Items	1	2	3	4	5
Age	75	68	45	55	62
Sex	Male	Male	Male	Female	Male
Durie-Salmon staging system	IIIA	IIIB	IIIA	IIIA	IIIB
International staging system	III	III	II	III	III
RISS stage	III	II	II	II	II
Extramedullary involvement	The liver is involved	None	None	None	Pulmonary involvement
Multiple lytic bone lesions (X-ray)	None	None	Positive	Positive	Positive
BM sample of Congo red Staining	Negative	Negative	Negative	Negative	Negative
BM biopsy Involvement of MM cells	Diffuse	Diffuse	Focal	Diffuse	Diffuse
Percentage of plasma cells in BM (Morphology)	73.5%	60.5%	46.5%	72.5%	35.5%
Percentage of plasma cells in BM (Flow cytometry)	12.9%^a^	8.2%^a^	4.1%^a^	3.3%^a^	0.4%
Chromosomal study	Complex chromosomal Abnormalities	45, X, -Y	Normal	46, XX, inv(9)(p12q13)	Complex chromosomal abnormalities
FISH	t(11;14)	Normal	Normal	Normal	t(11;14)
CBC					
WBC (× 10^9^/L)	3.57	6.54	3.86	3.52	10.63
HGB (g/dL)	2.7	7.0	8.3	11.9	8.3
PLT (×10^9^/L)	59	138	101	213	242
LDH (U/L) (100~ 250)	291	231	177	180	217
Serum calcium	Low	Low	Normal	Normal	Normal
Albumin (g/dL)	38	30	43	42	40
Serum β2 micoglobulin (mg/L)	11.39	27.63	4.06	7.40	10.14
Serum creatinine mg/dL	1.4	8.2	0.9	1.1	2.0
Serum Ig (mg/dL)					
IgD	364	Trace^b^	653	1063	394
IgA	16.3	35.7	15.5	4.87	21.4
IgG	523	545	427	1110	618
IgM	9.4	10.4	5.1	72.8	22
Serum light chain type	Lambda	Lambda	Lambda	Lambda	Lambda
Urine M protein (g/day)	5.80	0.31	6.25	3.95	0
Overall survival (month)	6	4	31	13	39
Living state	Dead	Dead	Alive	Dead	Alive

^a^Flow cytometry analysis was performed to these patients after chemotherapy

^b^The IgD was too low to detect. The serum IgD of this patient was detected after four cycles of chemotherapy

*BM* bone marrow, *MM* multiple myeloma, *Ig* immunoglobulin, *CBC* complete blood count, M protein, monoclonal protein

**Fig. 2:**
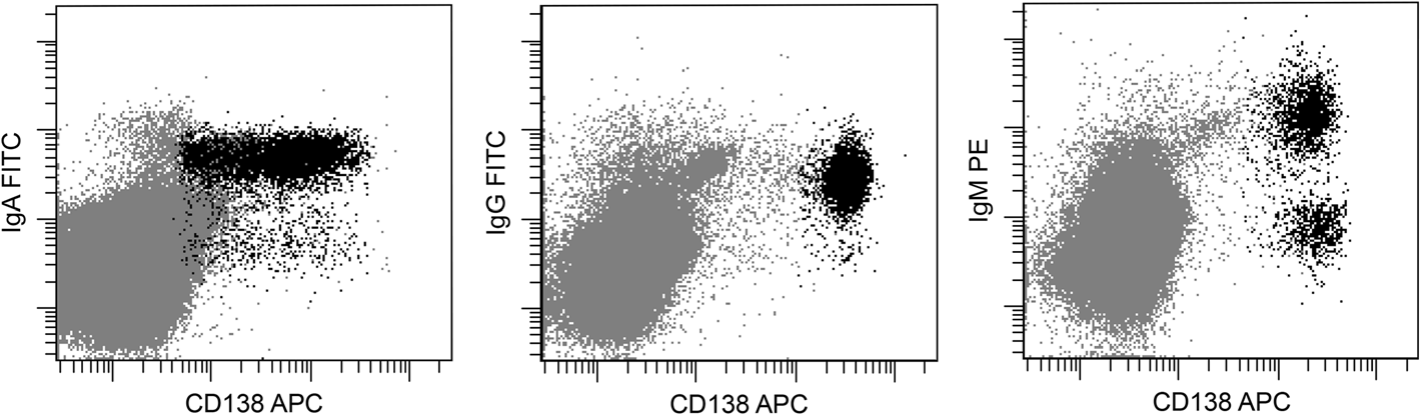
Flow cytometry results of MM patients with other heavy chain subtypes. Bone marrow samples of patients with IgA, IgG, or IgM MM were analyzed by flow cytometry. We chose one typical example of each subtype to display. The positive expression of cytoplasmic IgA, IgG, and IgM is shown from left to right

**Fig. 3:**
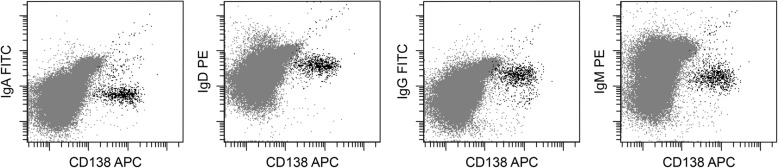
Flow cytometry results of MM patients with light-chain subtypes. Bone marrow samples of nine light-chain MM patients were analyzed by flow cytometry. The flow cytometry results of a representative patient are shown. The negative expression of cytoplasmic IgA, IgD, IgG, and IgM is shown from left to right

Diagnostic results obtained using flow cytometry and traditional methods are listed in Table 2. Among 9 IgG MM patients, the cytoplasmic IgG of 8 patients was detected by flow cytometry. The cytoplasmic IgM was detected in 1 patient with IgM MM, and the cytoplasmic IgA was detected in 10 patients with IgA MM, while no cytoplasmic heavy chain was detected in 9 patients with light chain MM and 1 patient with non-secretory MM. Expression of other heavy chains by flow cytometry in a particular type of heavy chain myeloma were negative. The flow cytometry results of cytoplasmic heavy chain of three patients with IgA, IgG, and IgM MM respectively are shown in Fig. 2. And the flow cytometry results of a patient with light-chain MM are shown in Fig. 3.

**Table 2:** Diagnostic result of MM patients by using flow cytometry and traditional methods

Subtype	Flow cytometry (n)	Traditional methods^a^ (n)	*P*
IgD	5	5	> 0.05
IgG	8	9	> 0.05
IgM	1	1	> 0.05
IgA	10	10	> 0.05
Light chain	9	9	> 0.05
Non-secretory	1	1	> 0.05

*MM* multiple myeloma, *IgD* immunoglobulin D, *IgG* immunoglobulin G, *IgM* immunoglobulin M, *IgA* immunoglobulin A

^a^The monoclonal immunoglobulin was detected by serum protein electrophoresis, urine protein electrophoresis; serum and urine immunofixation electrophoresis (IFE); serum and urine free light chain (FLC) assay. In this article, we named these methods as traditional methods

## Discussion

IgD MM is a rare type of MM due to its low prevalence and the low sensitivity of its diagnostic methods. According to other reports, lower M protein level is not uncommon in IgD MM [3, 14]. The serum M protein of higher than 2 g/dl was detected in only 14% of IgD MM patients [2], and the urine light chain on electrophoresis of higher than 4 g/d and 1 g/d was observed in 28% [2] and 61% [4] of IgD MM patients, respectively. A previous study showed a high sensitivity for detecting IgD in IgD MM patients using two-dimensional gel electrophoresis. However, the authors considered this technique time-consuming, difficult to perform, and expensive. Therefore, they would not recommend it as the first-line procedure to routinely diagnose IgD MM [15].

To evaluate whether detecting cytoplasmic IgD using flow cytometry could be a reliable supplemental method to diagnose IgD MM, we chose four patients with IgD MM after receiving chemotherapy and successfully detected positive expression of cytoplasmic IgD in residual MM cells from their BM samples (No. 1~ 4). We also evaluated a patient newly diagnosed with IgD MM (No. 5) and detected the IgD level using both flow cytometry and the traditional methods.

BM samples of four IgD MM patients (No. 1~ 4) were evaluated by flow cytometry after chemotherapy, while the percentages of malignant plasma cells in BM from the same patients were evaluated by morphology before treatment. Only one BM sample (No. 5) was concurrently analyzed by morphology and flow cytometry before chemotherapy. In the fifth patient, the BM was fully packed with MM cells. During BM collection, difficulty was experienced when aspirating a BM sample, and the sample was tremendously diluted by peripheral blood. This might be the reason that 35.5% of MM cells were detected by morphology, but only 0.4% of MM cells were detected by flow cytometry.

As shown in the results, the minimal proportion of IgD-positive MM cells detected by flow cytometry was 0.4%. This finding demonstrated that flow cytometry is a sensitive method to detect very low amounts of IgD MM cells.

Since detecting cytoplasmic IgD in patients with IgD MM has been worked so well, we want to answer the following questions: Can cytoplasmic IgD only be detected in IgD MM? In MM patients with IgA, IgG, or IgM subtype, can the corresponding cytoplasmic Ig heavy chain be detected in MM cells? How the expression levels of the four heavy chains in MM patients with light chain or non-secretory subtype? So we estimated the presence of the four immunoglobulin heavy chains in other MM subtypes. The diagnostic consistency between flow cytometry and traditional methods was 96.67% (29/30 patients). Only one IgG MM patient had a negative cytoplasmic IgG result based on flow cytometry analysis.

As for the technique itself, flow cytometry is easy to perform and suitable for routine clinical use.

## Conclusions

Detection of cytoplasmic IgD using flow cytometry could be a convenient, sensitive, and supplemental method to diagnose IgD MM. Analysis of additional samples is needed to certify the use of this technique in clinical applications. This technique might be recommended as a routine procedure in the future.

## Abbreviations

BM: Bone marrow
CBC: Complete blood count
FLC: Free light chain
IFE: Immunofixation electrophoresis
IgD: Immunoglobulin D
IMWG: International Myeloma Working Group
M protein: Monoclonal protein
mAbs: Monoclonal antibodies
MM: Multiple myeloma
SPEP: Serum protein electrophoresis
UPEP: Urine protein electrophoresis
